# Examining the association of career stage and medical specialty with personality preferences – a cross-sectional survey of junior doctors and attending physicians from various specialties

**DOI:** 10.1186/s12909-019-1789-2

**Published:** 2019-09-23

**Authors:** Yu-Che Chang, Hsu-Min Tseng, Xaviera Xiao, Roy Y. L. Ngerng, Chiao-Lin Wu, Chung-Hsien Chaou

**Affiliations:** 1 Chang Gung Medical Education Research Center (CG-MERC), Chang Gung Memorial Hospital, Linkou, Taiwan; 2 Department of Emergency Medicine, Chang Gung Memorial Hospital, Linkou, Taiwan; 3 Chang Gung University College of Medicine, Taoyuan, Taiwan; 4 Department of Health Care Management, Chang Gung University, Taoyuan, Taiwan; 5 Risk Society and Policy Research Center, National Taiwan University, Taipei, Taiwan; 6 Department of Otorhinolaryngology, Head and Neck Surgery, Chang Gung Memorial Hospital, Linkou, Taiwan

**Keywords:** Personality preference, Personality type, Clinical training, Myers–Briggs type Indicator, Cross-sectional survey, Career stage, Specialty

## Abstract

**Background:**

Personality preference research on medical students and physicians demonstrates that personality preferences may affect one’s choice of specialty and transform over the course of one’s academic career as well as during one’s time spent in the clinical setting. The literature offers valuable methods for evaluating medical curricula, understanding medical specialties, and rethinking communication techniques between educators and learners. In line with this encompassing body of work, this study examines the personality preferences of junior doctors and attending physicians from various specialties to investigate how career stage and medical specialty are associated with personality preferences.

**Method:**

The Myers–Briggs Type Indicator (MBTI) was applied to assess the personality preferences of junior doctors (postgraduates year 1–3) and attending physicians from six major medical specialties. Participants completed a self-administered 93-item questionnaire, while a certified MBTI practitioner explained the personality dichotomies as well as facilitated the self-evaluation process and the questionnaire’s interpretation. Contrasted dichotomous scores and radar plots were employed to illustrate the distinction between junior doctors and attending physicians’ personality preferences. All analyses were performed using the SAS statistical software, while a Wilcoxon rank-sum test was used to quantify the polarisation of personality preferences between junior doctors and attending physicians.

**Results:**

In total, 98 participants were recruited, of whom 59 were attending physicians and 39 were junior doctors. The most common personality types among the junior doctors were ESTJ (15.4%), INTP (12.8%), and ESFJ (10.3%), while among the attending physicians, the most common types were ISTJ (23.7%) and ESTJ (18.6%). Both junior doctors and attending physicians expressed personality preferences for sensing, thinking, and judging. However, compared to the junior doctors, more polarised personality preferences were noted among the attending physicians for sensing (*p* = 0.038), thinking (*p* = 0.032), and judging (*p* = 0.024). Moreover, junior doctors exhibited less distinct personality preferences in this study.

**Conclusion:**

Attending physicians and junior doctors exhibited greater personality inclinations for sensing, thinking, and judging, although the former expressed these personality preferences more strongly than the latter. These findings highlight that, amongst physicians, career stage is strongly associated with the expression of personality preferences.

## Background

Previous studies reveal distinct differences in the personality preferences among physicians and the general population—that is, physicians’ potential patients [1, 2]. Namely, medical students and physicians express greater personality preferences for thinking and judging [3–5]. Several studies have found that the personality preference for thinking is attributed to physicians in specialties such as surgery [6–8] and emergency medicine (EM) [2, 9, 10]. Even among healthcare executives, the preference for thinking was more pronounced than among their general business counterparts [11]. A greater understanding of physicians’ personality preferences can enhance their professional awareness [12] and, by extension, improve their approach to patient care [1]. Personality preferences also speak to physicians’ capacity for risk tolerance and shed light on their decision-making processes in the clinical setting [13, 14]. For instance, Higgs’s study reveals that the intuition preference is associated with higher levels of emotional intelligence [15]. The literature also demonstrates that a greater understanding of personality preferences may affect medical training curricula insofar as personality preferences can illuminate learners’ support needs and learning styles [4, 16]. Moreover, personality preference research has led to a more comprehensive understanding of how faculty evaluations of residents’ performances should be approached [17, 18].

Within research on physicians’ personality preferences, some opportunities have been overlooked. Relatively few studies interrogate the differences in personality preferences among physicians at different career stages and across various medical specialties. As such, our research aims to determine how career stage and medical specialty are associated with personality preferences by examining those preferences among junior doctors and attending physicians. Although many junior doctors in our study had nominated a specialty, their specialty choices were exclusively provisional and were therefore not considered; conversely, attending physicians’ specialty choices were considered an area of interest. We referred to previous findings on the personality preferences of emergency physicians (EPs), physicians from surgical specialties, and physicians from non-surgical specialties to determine whether or not similar trends may be identified among our study population.

## Methods

### Study design and setting

Our cross-sectional study utilised the Myers–Brigg Type Indicator (MBTI) instrument to assess the personalities of our participating junior doctors (post-graduate years 1–3) and attending physicians. The sample size was predetermined according to previous studies wherein the MBTI was deployed [19, 20]. Convenience sampling was used, and the study was conducted between August 1, 2015 and July 31, 2016 at Chang Gung Memorial Hospital, Linkou branch—a large, 3800-bed, tertiary, private, multispecialty medical centre located in an urban area in North Taiwan. Ethical approval for this study was obtained from the Chang Gung Memorial Hospital and Chang Gung University Institutional Review Board (103–7538B). Participation was voluntary, informed consent was acquired, and participants’ anonymity was guaranteed.

### Participants

Attending physicians from internal medicine, paediatrics, surgery, obstetrics, and gynaecology (OB/GYN), EM, and family medicine were recruited. These six key medical specialties were chosen because they tend to encompass a high proportion of attending physicians. Notably, these six specialties are among the top nine in the United States in terms of the number of physicians employed therein [21]. These specialties encompass 66% of all working physicians in the United Kingdom [22] and 72% in Australia [23]. In Taiwan, these medical specialties are similarly prominent, and all medical graduates are in fact required to participate in a clinical rotation curriculum in the aforementioned specialties [24]. When considering the prominence of these six medical specialties, their inclusion in our sample of attending physicians was an important element of building a representative sample of attending physicians’ personality preferences. Hereafter, these specialties will be referred to as surgical specialties (i.e., surgery and OB-GYN), EM, and non-surgical specialties (i.e., family medicine, paediatrics, and internal medicine). By differentiating between surgical, non-surgical, and EM, our study attempts to consider whether or not any distinctions between attending physicians may be noted. Although junior doctors make provisional specialty choices, these provisional choices are not considered in our research.

### Instrument and data collection

Developed in 1962, the MBTI questionnaire is a personality assessment tool based on Carl Jung’s theory of psychological types. The questionnaire has been used extensively for the purposes of career assessment and leadership development as well as to understand communication approaches, learning styles, counselling orientations, and methods of coaching and teambuilding [19, 25]. The MBTI instrument’s value and use in psychotherapy, the clinical setting, career development, and research settings are based on its reliability and validity [26]. The MBTI comprises four dichotomies of personality preferences: *extraversion/introversion* (E/I); *sensing/intuition* (S/N); *thinking/feeling* (T/F); and *judging/perceiving* (J/P) [27]. These four axes form sixteen different combinations, such as ESTJ (extroverted, sensing, thinking, judging) or INFP (introverted, intuition, feeling, perceiving), that are also known as personality types. A detailed description of these dimensions is summarised in Table 1 [27]. Despite the clarity of these dichotomies, Lloyd [28] cautions that the personality preferences within each of the MBTI’s four dimensions should not be perceived as wholly distinct; namely, an individual is constitutionally neither an extrovert nor an introvert.

**Table 1:** Summary of MBTI four pairs of dichotomies of preferences

Dichotomy	Description
Extraversion (E) – Introversion (I)	Look at whether people prefer to focus their perceptions and judgment on the outer (E) or inner (I) worlds
Sensing (S) – Intuition (N)	Look at whether people absorb information by observing facts using their five senses (S) or via using their intuition and looking at meanings (I)
Thinking (T) – Feeling (F)	look at whether people logical think through their decisions (T) or rely on their feelings and values to make decisions (F)
Judging (J) – Perceiving (P)	look at whether people deal with the outer world using a judgment (J) or perceptive (P) process.

Myers, I., & Myers, P. (2010). Gifts differing: Understanding personality type. Nicholas Brealey Publishing

We engaged CCP Asia Pacific—which has been certified by the Myers and Briggs Foundation—to administer the MBTI instrument to our participants. A certified MBTI practitioner facilitated the administration and interpretation process, starting with an introduction to the four dichotomies and an explanation of the sixteen personality types. Participants self-evaluated their personality preferences before filling out the 93-item MBTI questionnaire and were rated on each dimension to produce their four-letter personality types. The MBTI results were delivered via a conversation facilitated by a trained practitioner who clarified any ambiguity regarding the MBTI typology and helped the respondents reach a consensus for their personality types.

### Statistical analysis

All analyses were performed using the SAS statistical software. Numerical data were presented as the mean (standard deviation, SD), and categorical data for the personality preference distribution were presented as the count (percentage). A comparison of contrasted dichotomous scores on individual MBTI profiles between the junior doctors and attending physicians was made using a Wilcoxon rank-sum test to quantify the polarisation of personality preferences between the two groups. Radar plots were also created to illustrate the differences in the MBTI results between the junior doctors and attending physicians from various specialties.

## Results

### Participants’ characteristics

The study involved 59 attending physicians, including 23 non-surgical physicians (12 internists, 6 paediatricians, and 5 family medicine physicians), 18 EPs, and 18 physicians from surgical specialties (14 surgeons and 4 OB-GYNs). The attending physicians comprised 45 males and 14 females, with a mean age (±SD) of 41.6 ± 6.4 years, thus averaging 9.92 ± 5.71 years of experience. Of the 39 junior doctors, 32 were in their post-graduate year 1 (PGY1) and 7 were in their post-graduate year 2–3 (PGY2–3). Amongst the 39 junior doctors, 25 were male and 14 were female, and the mean age was 27.6 ± 1.77 years.

### Distribution of participants’ personality preferences

A detailed list of scores for each personality type for the various participant groups is provided in Table 2. With the exception of the E/I dimension, the mean score difference between the two personality types in each dichotomy was distinct between the junior doctors and attending physicians. The most notable difference was the J/P dichotomy, which grew from 2.67 among junior doctors to 9.08 among attending physicians.

**Table 2:** Descriptive and demographic results

	Junior Doctors^a^		Attending physicians							
	(*n* = 39)		Overall (*n* = 59)		Non-surgical^b^ (*n* = 23)		Surgical (*n* = 18)		EM (*n* = 18)	
	mean	(SD)	mean	(SD)	mean	(SD)	mean	(SD)	mean	(SD)
Gender										
M (%)	25	(64.1)	45	(76.3)	16	(69.6)	15	(83.3)	14	(77.8)
F (%)	14	(35.9)	14	(23.7)	7	(30.4)	3	(16.7)	4	(22.2)
Age (yrs)	27.6	(1.77)	41.6	(6.4)	44.0	(6.92)	40.6	(5.09)	39.6	(6.20)
Seniority (yrs)	NA		9.92	(5.71)	11.6	(6.47)	8.78	(4.26)	8.89	(5.70)
MBTI results										
Extrovert	9.90	(6.91)	9.31	(5.95)	8.35	(6.24)	10.22	(6.23)	9.61	(5.44)
Introvert	11.0	(6.94)	11.7	(5.95)	12.65	(6.24)	10.78	(6.23)	11.39	(5.44)
Sensing	14.5	(4.96)	16.7	(4.99)	17	(4.83)	17.44	(5.14)	15.61	(5.14)
Intuition	11.5	(4.96)	9.24	(4.95)	8.91	(4.79)	8.56	(5.14)	10.33	(5.08)
Thinking	12.2	(5.41)	14.8	(5.47)	13.61	(5.85)	15.94	(4.87)	15.28	(5.51)
Feeling	11.8	(5.41)	9.15	(5.47)	10.39	(5.85)	8.06	(4.87)	8.67	(5.52)
Judging	12.3	(6.94)	15.5	(6.27)	16.87	(6.70)	14.28	(6.09)	15.11	(5.89)
Perceiving	9.67	(6.94)	6.46	(6.27)	5.13	(6.70)	7.72	(6.09)	6.89	(5.89)
Dichotomy										
I (I, %)	22	(56.4)	32	(54.2)	14	(60.9)	7	(38.9)	11	(61.1)
II (S, %)	23	(59.0)	44	(74.6)	18	(78.3)	15	(83.3)	11	(61.1)
III (T, %)	22	(56.4)	36	(61.0)	12	(52.2)	14	(77.8)	10	(55.6)
IV (J, %)	20	(51.3)	42	(71.2)	18	(78.3)	12	(66.7)	12	(66.7)

^a^PGY1–3

^b^EM physician was not included in non-surgical physicians

*Abbreviation*: *EM* Emergency medicine, *NA* Non-accessible

At the bottom of Table 2, the participants were categorised according to their higher scores for each personality dichotomy. Overall, junior doctors and attending physicians were more likely to express ISTJ. However, in comparison to junior doctors, attending physicians expressed stronger preferences for the latter three (sensing, thinking, and judging). The frequency distribution for the personality type combinations is presented in Table 3. The most common personality types were ESTJ (15.4%), INTP (12.8%), and ESFJ (10.3%) among the junior doctors and ISTJ (23.7%) and ESTJ (18.6%) among the attending physicians.

**Table 3:** Distribution of personality preference

		Junior Doctors (*n* = 39)				Attending Physicians (*n* = 59)			
		Sensing (S)		Intuition (N)		Sensing (S)		Intuition (N)	
		Thinking (T)	Feeling (F)	Thinking (T)	Feeling (F)	Thinking (T)	Feeling (F)	Thinking (T)	Feeling (F)
Introvert (I)	Judging (J)	ISTJ 3 (7.69)	ISFJ 0 (0.00)	INTJ 2 (5.13)	INFJ 3 (7.69)	ISTJ^a^ 14 (23.7)	ISFJ 5 (8.47)	INTJ 2 (3.39)	INFJ 2 (3.39)
	Perceiving (P)	ISTP 3 (7.69)	ISFP 3 (7.69)	INTP^a^ 5 (12.8)	INFP 1 (2.56)	ISTP 1 (1.69)	ISFP 3 (5.08)	INTP 2 (3.39)	INFP 3 (5.08)
Extrovert (E)	Perceiving (P)	ESTP 2 (5.13)	ESFP 2 (5.13)	ENTP 2 (5.13)	ENFP 2 (5.13)	ESTP 2 (3.39)	ESFP 3 (5.08)	ENTP 1 (1.69)	ENFP 2 (3.39)
	Judging (J)	ESTJ^a^ 6 (15.4)	ESFJ^a^ 4 (10.3)	ENTJ 0 (0.00)	ENFJ 1 (2.56)	ESTJ^a^ 11 (18.6)	ESFJ 5 (8.47)	ENTJ 2 (3.39)	ENFJ 1 (1.69)

Data presented as number (%)

^a^proportion greater than 10%

When breaking down the specialties, attending physicians from surgical specialties stood out among the attending physicians in the E/I and T/F dimensions. Attending physicians from surgical specialties (77.8%) expressed stronger preferences for thinking, while the attending physicians from non-surgical specialties (52.2%) and EPs (55.6%) had broadly similar preferences for thinking as did the junior doctors (56.4%). Additionally, junior doctors, attending physicians from non-surgical specialties, and EPs were more likely to be introverted (56.4–61.1%), while attending physicians from surgical specialties were more likely to be extroverted (61.1%). Although all attending physicians exhibited a greater preference for sensing, EPs demonstrated the lowest preference (61.1%) compared to attending physicians from both surgical specialties (83.3%) and non-surgical specialties (78.3%). Lastly, while all attending physicians exhibited a high preference for judging, physicians from non-surgical specialties (78.3%) expressed a higher preference for judging than did the EPs (66.7%) and physicians from surgical specialties (66.7%).

### Polarisation of personality preferences between junior doctors and attending physicians

**Fig. 1:**
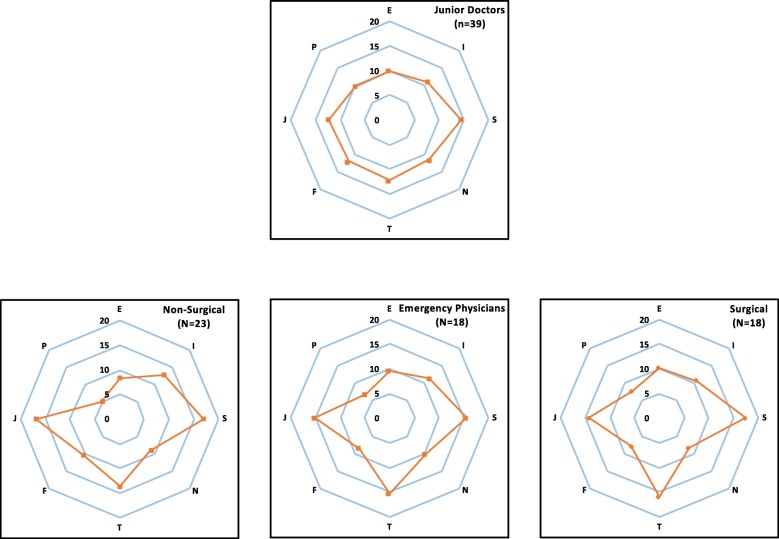
Radar plot of attending physicians’ MBTI profiles according to specialties. A more circular shaped figure was presented in the junior doctors’ plot, indicating more balanced personality preferences, as compared to the attending physicians’ plots which contain multiple spikes

The differences in the junior doctors’ and attending physicians’ scores for each dimension are analysed and depicted in Table 4. The findings reveal a trend of polarisation within three personality dichotomies; the mean difference in the attending physicians’ scores was significantly greater than that of the junior doctors in dichotomy II–S/N (7.47 vs 2.92, *p* = 0.038), dichotomy III–T/F (5.68 vs 0.41, *p* = 0.032), and dichotomy VI–J/P (9.08 vs 2.67, *p* = 0.024). These results indicate that, in our cross-sectional survey, junior doctors possess notably balanced personality preferences, while attending physicians have more pronounced preferences for sensing, thinking, and judging. In Fig. 1, radar plots stratified by specialties for the junior doctors and attending physicians illustrate the distinction between the personality preferences of both. A more circular-shaped figure was presented in the junior doctors’ plot, which indicates relatively balanced personality preferences. This result stands in contrast against the attending physicians’ plot, which contains multiple spikes and demonstrates that attending physicians express the same personality preferences as their counterparts albeit to a greater degree.

**Table 4:** Comparisons of contrasted dichotomous scores^a^ on MBTI profiles between junior doctors and attending physicians

	Attending physicians		Junior Doctors		*p*-value
Dichotomy I (Introvert - Extrovert)	2.39	(11.9)	1.21	(13.8)	0.727
Dichotomy II (Sensing - Intuition)	7.47	(9.94)	2.92	(9.91)	0.038*
Dichotomy III (Thinking - Feeling)	5.68	(10.9)	0.41	(10.8)	0.032*
Dichotomy IV (Judging - Perceiving)	9.08	(12.5)	2.67	(13.9)	0.024*

Data presented as mean (SD)

^a^Contrasted dichotomous scores, namely the difference of two aspect of score measurements within the same dichotomy, between junior doctors and attending physicians

*Statistically significant (*p* < 0.05) using independent Wilcoxon rank-sum test

## Discussion

Our study determined that junior doctors and attending physicians from different specialties are most likely to exhibit the sensing, thinking, and judging preferences, thus reaffirming what has been identified in previous research [3–5, 29, 30]. More importantly, this study makes a valuable contribution to the medical field by revealing that, in three of four personality preferences (sensing, thinking, and judging), the attending physicians expressed stronger personality preferences than did the junior doctors. The latter were remarkably more neutral in their preferences for sensing, thinking, and judging, thus indicating a statistically significant difference.

This finding suggests that clinical experience may be a factor in the personality preferences of junior doctors and attending physicians alike, which reflects prior research on personality preferences and their development. Medical and dental students have been tested with the MBTI to demonstrate that students’ personality preferences change over time [31–33], while other research identifies that teachers possess more polarised personality preferences than their students [18]. Considering the impact of clinical experience is important in any study that tackles personality preferences insofar as personality and its development can be affected by major or minor life events. Notably, work-related events have a significant effect in changing the behavioural or cognitive traits of one’s personality [34]. As Martinou’s [35] work reveals, junior trainees are likely to enjoy human interaction and may therefore exhibit a greater preference for feeling; however, as their careers progress, they may begin expressing a greater preference for thinking as they reflect upon their practice. Indeed, physicians are trained to use scientific evidence when exercising their judgment, and clinical experiences allow physicians to refine their critical thinking and decision-making skills, which may accentuate their thinking and judging personality preferences.

In addition to comparing junior doctors and attending physicians, our study makes a unique contribution to medical field by considering how one’s specialty choice might play a role in one’s personality preferences. As is illustrated in Fig. 1, our study considers how attending physicians across key medical specialties (i.e., physicians from surgical specialties, EPs, and physicians from non-surgical specialties) may differently emphasise certain personality preferences. To our knowledge, ours is the first study to incorporate attending physicians from various specialties and junior doctors. Several personality assessment tools have been implemented to explore the relationship between personality preferences and medical specialty amongst undergraduates in order to facilitate tailored career coaching and help students evaluate the many existing specialties [7, 8]. Bell [18] examines the diverse personality styles of post-graduate medical students and their teachers as well as their impact on learning. To our knowledge, large-scale studies concerning personality preferences in healthcare focus on medical students [17], and very few have been conducted with clinical teachers, senior physicians, or other medical staff [36, 37]. Although the case number of enrolled physicians from various specialties is relatively limited in our research, we identified interesting trends that are noteworthy for future studies.

Compared to EPs and attending physicians from non-surgical specialties, attending physicians from surgical specialties expressed the strongest preferences for extraversion and thinking. This result affirms studies wherein the MBTI and alternative personality assessment tools reveal similar findings [7, 8, 38, 39]. The overall consistency of surgeons’ personality preference for extraversion suggests that it is worthwhile to consider how surgical training in their work context might require extraversion; additional thorough research might inform how such a work culture affects surgeons who express a greater personality preference for introversion. The EPs in our study were more likely to exhibit the ITSJ preferences; with the exception of the E/I profile, the main difference between EPs and attending physicians from surgical and non-surgical specialties was highlighted in the S/N dichotomy. Although all attending physicians in our study expressed a preference for sensing, EPs exhibited less preference for sensing (61.1%) than did attending physicians from both surgical specialties (83.3%) and non-surgical specialties (78.3%). In other words, the characteristics of an intuitive personality preference are not diametrically oppositional in understanding how EPs process information. Within Boyd and Brown’s study of EPs’ personality types, the single most common personality preference in their study cohort was the ENTJ type (*n* = 12; 17.7%); in fact, 58.8% of the EPs in the study exhibited the intuitive trait [2]. One possible explanation for this finding may be that, as outlined in the 2016 Model of the Clinical Practice of Emergency Medicine [40], when patients in the emergency department present ambiguous symptoms rather than a known illness or disease, EPs must use pattern recognition to diagnose conditions during such time-constrained encounters [41]. Paying attention to patterns and deconstructing their meanings are tasks associated with intuition [27]. Moreover, multitasking—a core skill in EM—requires the intuitive trait of pattern recognition [40, 42].

In our study cohort, attending physicians from non-surgical specialties were more likely to exhibit the judging (78.3%) preference than the attending physicians from surgical specialties (66.7%) and EPs (66.7%). Making decisions in a planned and organised manner with as much control as possible is a hallmark of attending physicians from non-surgical specialties. By contrast, EPs and attending physicians from surgical specialties may prefer flexibility and spontaneity in their decision making due to the nature of their work [27, 43, 44]. Indeed, we identified that attending physicians from surgical specialties and EPs scored higher in tough-mindedness and in impulsive sensation seeking than family medicine residents; the former were more likely to be ‘adrenaline junkies’ who expressed greater stress resistance and risk-taking abilities [42, 45, 46]. Overall, a scarce number of studies investigate the personality preferences of attending physicians from non-surgical specialties, and thus future research must be conducted to reveal more favourable comparisons and to construct a greater understanding of physicians from heavily staffed non-surgical specialties.

### Limitations

The MBTI instrument is very costly. Due to limited funding, this study included merely 98 participants. Therefore, whilst we were also able to observe a trend in how attending physicians from surgical specialties, non-surgical specialties, and EM differed in their personality preferences, the sample size for each group was relatively small. Nevertheless, our findings permitted us to make connections to the personality preference literature that specifically explores specialisation. The limited number of participants also indicated that directly examining any personality type would be inappropriate; examining personality types is especially difficult because the MBTI personality assessment tool constitutes sixteen possible personality types. Financial parameters also led us to employ convenience sampling, and although it served our aims, we recognise that this approach carries a degree of bias. Finally, this study was conducted in a single medical centre. Further studies that utilise the MBTI instrument and include a greater number of attending physicians and junior doctors across various hospitals may strengthen our understanding of personality preferences among both junior doctors and attending physicians from various specialties.

## Conclusion

Our study demonstrates that junior doctors and attending physicians exhibit personality preferences for sensing, thinking, and judging. Attending physicians express these personality preferences more strongly than do junior doctors, and these findings highlight that, amongst physicians, career stage is associated with the accentuation of personality preferences. Furthermore, a more thorough understanding of personality preferences amongst physicians at varying career stages and across specialties can promote greater self-awareness amongst physicians as they negotiate workplace challenges and seek to improve their communication skills.

## Data Availability

The datasets used and/or analysed during the current study are available from the corresponding author on reasonable request.

## Appendix

**Table 5:** Career stage and specialty group of participants

Career stage	Specialty group	No.
Junior doctors		39
PGY1^a^	NA^c^	32
PGY2	Psychiatry	1
	Emergency Medicine	1
PGY3	Nuclear Medicine	1
	Laboratory Medicine	1
	Family Medicine	2
	Physical Medicine and Rehabilitation	1
Attending Physicians^b^		59
	Surgery	14
	Obstetrics and Gynecology	4
	Emergency Medicine	18
	Pediatrics	6
	Family Medicine	5
	Internal medicine	12

^a^Abbreviation: PGY1, postgraduate year 1

^b^Qualified specialist, attending hospital in particular specialty

^c^PGY1 Participants make provisional specialty choices

## Abbreviations

E/I: Extraversion/introversion
EM: Emergency medicine
EP: Emergency physician
ESTJ: Extraversion, sensing, thinking, judging
INTP: Introversion, intuition, thinking, perceiving
J/P: Judging/perceiving
MBTI: Myers–Briggs Type Indicator
PGY1: Post-graduate year one
S/N: Sensing/intuition
T/F: Thinking/feeling
